# Alpha-1-antitrypsin antagonizes COVID-19: a review of the epidemiology, molecular mechanisms, and clinical evidence

**DOI:** 10.1042/BST20230078

**Published:** 2023-06-09

**Authors:** Xiyuan Bai, Tony Schountz, Ashley M. Buckle, Janet L. Talbert, Robert A. Sandhaus, Edward D. Chan

**Affiliations:** 1 Department of Medicine, Rocky Mountain Regional Veterans Affairs Medical Center, Aurora, CO, U.S.A.; 2 Department of Academic Affairs, National Jewish Health, Denver, CO, U.S.A.; 3 Division of Pulmonary Sciences and Critical Care Medicine, University of Colorado School of Medicine, Aurora, CO, U.S.A.; 4 Department of Microbiology, Immunology, and Pathology, Colorado State University, Fort Collins, CO, U.S.A.; 5 Department of Biochemistry and Molecular Biology, Biomedicine Discovery Institute, Monash University, Clayton, Victoria, Australia; 6 PTNG Bio, Melbourne, Australia; 7 Department of Pediatrics, Vanderbilt University Medical Center, Nashville, TN, U.S.A.; 8 Department of Medicine, National Jewish Health, Denver, CO, U.S.A.

**Keywords:** alpha-1-antitrypsin, COVID-19, heparin, SARS-CoV-2, serine protease, serpin

## Abstract

Alpha-1-antitrypsin (AAT), a serine protease inhibitor (serpin), is increasingly recognized to inhibit SARS-CoV-2 infection and counter many of the pathogenic mechanisms of COVID-19. Herein, we reviewed the epidemiologic evidence, the molecular mechanisms, and the clinical evidence that support this paradigm. As background to our discussion, we first examined the basic mechanism of SARS-CoV-2 infection and contend that despite the availability of vaccines and anti-viral agents, COVID-19 remains problematic due to viral evolution. We next underscored that measures to prevent severe COVID-19 currently exists but teeters on a balance and that current treatment for severe COVID-19 remains grossly suboptimal. We then reviewed the epidemiologic and clinical evidence that AAT deficiency increases risk of COVID-19 infection and of more severe disease, and the experimental evidence that AAT inhibits cell surface transmembrane protease 2 (TMPRSS2) — a host serine protease required for SARS-CoV-2 entry into cells — and that this inhibition may be augmented by heparin. We also elaborated on the panoply of other activities of AAT (and heparin) that could mitigate severity of COVID-19. Finally, we evaluated the available clinical evidence for AAT treatment of COVID-19.

## Introduction

There is burgeoning evidence that alpha-1-antitrypsin (AAT), the most abundant endogenous serine protease inhibitor (serpin), inhibits SARS-CoV-2 infection and mitigates many of the pathogenic mechanisms of COVID-19. Herein, we review the literature and mechanisms by which AAT antagonizes SARS-CoV-2 and COVID-19.

## Basic mechanism of SARS-CoV-2 infection

Upon binding of the spike protein of SARS-CoV-2 to its receptor angiotensin converting enzyme 2 (ACE2) on the cell membranes, the spike protein is sequentially cleaved by the cell surface serine proteases furin at the S1/S2 site and transmembrane protease 2 (TMPRSS2) at the S2’ site [1,2]. Moreover, neutrophil elastase, the canonical target of AAT, also cleaves near the S1/S2 site for the SARS-CoV-2 variant with the D614G mutation in the spike protein [3]. These modifications of the spike protein enable fusion of the viral envelope with the cell plasma membrane.

## COVID-19 remains problematic due to viral evolution and measures to prevent severe COVID-19 teeters on a balance

SARS-CoV-2, the cause of COVID-19, is the third beta-coronavirus and second sarbecovirus to emerge in humans in this century. It is likely that additional coronaviruses will jump the species barrier, potentially leading to new pandemics. Hence, despite the availability of efficacious COVID-19 vaccines, future SARS-related strains and other coronaviruses are likely to remain problematic because: (i) vaccine-refusal within a segment of the population, estimated to occur in 25–30% of the U.S. population per 2021 Gallup Poll and Monmouth University Polling Institute as well as the 4% vaccination rate in resource-poor countries, seedbeds for development of new SARS-CoV-2 variants; (ii) breakthrough cases even in those who have been vaccinated, especially in immunocompromised individuals [4–9]; (iii) fatigue in public health measures; (iv) anti-viral agents Paxlovid (nirmatrelvir + ritonavir) and molnupiravir were largely studied in the context of earlier SARS-CoV-2 strains with more recent occurrence of breakthrough symptoms and viral replication [10,11]; and (v) evolution of new SARS-CoV-2 strains such as the now dominant omicron strains (e.g. BQ.1, BQ.1.1, and XBB.1.5), which are much more transmissible, more immune evasive, less responsive to vaccines/boosters with protection that declines more rapidly, and/or increased or complete resistance to existing monoclonal antibodies [12–21]. Even as recently as September 2022, when the number of patients hospitalized for COVID-19 were declining, 500–1000 individuals still die each day (182 000–365 000 per year) from COVID-19 in the U.S. In contrast, 12 000–52 000 people die from influenza each year in the U.S. for the last 10 years before COVID-19.

## Treatment for severe COVID-19 remains suboptimal

Pneumonia/acute lung injury with refractory hypoxemia and septic shock due to a hyperinflammatory response — due largely to increases in inflammatory cells recruited and activated by interleukin-1 (IL-1), IL-6, IL-8, IL-17, and tumor necrosis factor (TNF) — are the most common causes of COVID-19 deaths [22–26]. Severe COVID-19 is also associated with CD4^+^ and CD8^+^ T cell lymphopenia and decreased interferon-gamma (IFNγ)-positive CD4^+^ T cells [27]. The current treatment for severe COVID-19 is largely supportive care combined with the glucocorticoid dexamethasone, which reduced mortality by 2.9% in those receiving supplemental oxygen only and by 12.1% in those requiring mechanical ventilation [28]. Remdesivir is also used for hospitalized patients [29] although its efficacy is controversial [30,31]. Anakinra (IL-1 receptor antagonist, IL-1Ra) has been evaluated for treatment of severe COVID-19 with no or marginal success [32–36].

Antagonists to IL-6 and IL-6 receptor (IL-6R) have shown either negative results or benefit in some patients recalcitrant to dexamethasone [37]. Several concerns with the use of antagonists to IL-6 or IL-6R are that IL-6 helps induce antibody production and activates T cells against infectious agents [22,38]. The FDA has also issued a Black Box warning for the anti-inflammatory Janus kinase 1 inhibitors (baricitinib, tofacitinib, upadacitinib) due to risks of serious cardiac events and cancer.

## Epidemiologic and clinical evidence that AAT deficiency increases risk of COVID-19 infection and more severe disease

AAT is the third most abundant protein in plasma and the most prevalent serpin in the body. As an acute phase protein, AAT levels increase three- to five-fold with systemic infection or inflammation, underscoring its important homeostatic function [39,40]. Persons with a normal *SERPINA1* gene are designated as protease inhibitor MM (PiMM). Two of the most common abnormal AAT alleles are Z-AAT (rs28929474) and S-AAT (rs17580), with the PiZZ, PiSZ, and some PiMZ genotypes resulting in severe or relative AAT deficiency. Some AAT alleles (F and I) encode a dysfunctional AAT protein but with normal circulating levels.

It has been estimated that approximately 100 000 individuals in the U.S. have severe AAT deficiency, with <10% currently identified [41]. However, this assessment is likely to be underestimated based on the calculation that if there are 13 million subjects with chronic obstructive pulmonary disease (COPD) in the U.S. — and that AAT deficiency (mostly PiZZ) is a well-established risk factor for COPD and PiZZ comprises 1–3% of all COPD cases [42] with both lower and higher prevalence reported based on the cohort defined [43] — then 130 000–390 000 with known COPD are predicted to be AAT deficient (PiZZ). The number of AAT deficient individuals in the U.S. population is likely to be even more as some patients with AAT deficiency have not developed COPD (e.g. children), COPD has yet to be detected in them, or have undiagnosed AAT deficiency-associated liver disease. Even in the absence of frank AAT deficiency, the AAT response to a systemic infection may be inadequate as has been shown for hospitalized COVID-19 patients [44,45] and among millions with certain heterozygous AAT mutations (PiMZ) [39]. Furthermore, oxidation of methionine 351 and/or 358 of even normal AAT may cause loss of its serpin activity [46]. Oxidation may also induce AAT polymerization [47,48]. Because COVID-19 is associated with increased oxidative stress [49], AAT may be rendered ineffective even with increased levels. Indeed, the concept of ‘relative AAT deficiency’ has been raised wherein external stressors such as oxidative stress, smoke exposure, high glucose levels, and bacterial proteases inactivates normal AAT [50].

COVID-19 cases were increased in parts of Italy with greater prevalence of AAT deficiency [51]. AAT-deficient subjects were also found to be 8.8-fold more likely to have symptomatic COVID-19 than the general population [52]. Shapira et al*.* [53] found a significant direct correlation between the frequency of the PiZ and PiS alleles with COVID-19 death rates in 67 countries. Yoshikura [54] also reported a robust correlation between the Pi*Z variant and the number of COVID-19 cases (correlation coefficient (CC) = 0.8584) and deaths (CC = 0.8713) in 68 countries. Both Europeans and Latinos have relatively higher rates of PiZZ, PiSZ, and PiMZ, estimated to be ∼12%, and are also the predominant ethnic groups in countries having the highest COVID-19 cases and deaths [55–58]. Furthermore, evidence implicates AAT deficiency itself — and not its pulmonary sequelae of emphysema or bronchiectasis – as a major risk factor for SARS-CoV-2 infection [57]. Hospitalized patients with severe COVID-19 had significantly lower plasma AAT levels than those admitted for non-COVID-19 pneumonia [59]. In a multi-center study of over 2000 patients with COVID-19, possessing the Pi*Z allele and/or plasma AAT level <116 mg/dl were highly associated with severe COVID-19 compared with non-severe disease [60]. In 105 subjects with AAT deficiency and COVID-19 infections in 10 European countries, poor outcome was more likely with PiZZ genotype than PiSZ; interestingly, only 14 subjects were receiving regular AAT augmentation and they showed a trend toward better outcome [61]. Murgia et al*.* [62] analyzed the effect of combined AAT anomalies (PiMZ, PiSZ, and PiMS) and air pollution on COVID-19 using the AAT*air pollution global risk score. They found that the ranking of the AAT*air pollution global risk score paralleled with the ranking of countries stratified by incidence of COVID-19 deaths [62].

McElvaney et al*.* [63] found the IL-6:AAT ratio is markedly elevated in critically ill COVID-19 patients compared with controls and that this ratio directly correlated with prolonged hospital stay and mortality. Since this study did not specifically target AAT-deficient subjects, it suggests that relatively normal or even modestly elevated AAT levels in the face of a severe infection may be wholly inadequate. Similarly, a study of 137 hospitalized patients with COVID-19 (of whom 56 were critically ill) found that the AAT:IL-6 ratio (reverse ratio from aforementioned study) was lower in non-survivors and those with thrombosis [64]. In COVID-19-associated acute respiratory distress syndrome, manifested by increased neutrophil elastase activity, there is an excess of protease:anti-protease ratio [65]. Interestingly, IL-6 induced AAT and tocilizumab (an IL-6R antagonist) down-regulated AAT in COVID-19 [65].

In contrast with the aforementioned studies, a community-based cohort study from the U.K. showed there was no increase in either SARS-CoV-2 infection or death rates in the presence of S- or Z-AAT alleles [66]. Similarly, in 61 Swedish patients hospitalized with COVID-19, only two were found to be PiMZ and none for PiMS, PiSZ, PiSS, or PiZZ [67]. While it is difficult to reconcile these incongruent findings between AAT anomalies and hospitalized COVID-19 cases, one plausible explanation is that some with known AAT deficiency took greater precautions as evinced by the finding that the AlphaNet (AAT-deficient) population had lower case rate but greater hospitalization rate for COVID-19 during the survey period from January 2020 to February 2022 [68]. In a large genome-wide association study (GWAS) meta-analysis of over 125 000 COVID-19 cases and 2.5 million controls, *SERPINA1* (AAT) gene polymorphism was not identified to be a major associative factor for severe COVID-19 [69]. However, the benefits of GWAS are well known but the limitations often overlooked. GWAS studies use power in numbers for complex and common diseases that have minor allele frequencies (MAF), typically >5% for common variants with small effects across large numbers of unrelated individuals; in contrast, genes with rare variants such as *SERPINA1* with MAF <5% typically associated in related individuals with large effects would be missed. The variants in GWAS themselves are not always the causal variant but may be signal for a nearby functional variant. Additional limitations of GWAS include the epidemiological design and the methodology (SNP arrays with imputation vs. whole genome sequencing) where SNP arrays may not be designed to capture genes with rare variants [70,71]. In summary, uncommon and rare genetic variants — as illustrated by *SERPINA1* genotypes that cause AAT deficiency — are not well captured by GWAS arrays [72,73]. Indeed, although one study found that the Z allele of *SERPINA1* gene was associated with COPD with an odds ratio of 1.17–1.74 [74], seven other GWAS or exome-wide studies did not find an association between COPD and the Z allele [75-81]; yet, the Z-AAT allele is strongly linked clinically to COPD [82]. In other words, if AAT deficiency had not been fortuitously identified in 1963, it is unlikely that GWAS or exome-wide studies would have identified *SERPINA1* variants (causing AAT deficiency) as a significant risk/associative factor for COPD.

## AAT inhibits TMPRSS2 and SARS-CoV-2 entry into cells

It was reported 30 years ago, in the context of HIV infection, that an engineered AAT (α1-PDX) inhibits furin, the serine protease that cleaves the spike protein of SARS-CoV-2 before cleavage by TMPRSS2 [83,84]. Five years *before* the COVID-19 pandemic, AAT was reported to inhibit TMPRSS2, also required to activate the hepatitis C virus [85]. Following binding to their cell surface receptors, the coronaviruses capable of causing fatal disease — with SARS-CoV and SARS-CoV-2 binding ACE2 and MERS-CoV binding DPP4 — all require TMPRSS2 to process the viral spike protein to gain cellular entry.

Because AAT is a potent serpin, it has the potential to inhibit both TMPRSS2 and SARS-CoV-2 infection. Indeed, in HEK293T cells engineered to overexpress TMPRSS2, physiologic concentrations of AAT potently inhibited TMPRSS2 activity [86,87]. Ritzmann et al. [88] showed in both two-dimensional submerged airway epithelial cells and three-dimensional airway epithelial organoid cultures that physiologic concentrations of AAT (1 and 5 mg/ml) significantly inhibited TMPRSS2 activity and decreased intracellular burden of SARS-CoV-2 [88]. Wettstein *et al*. [89] also demonstrated that AAT inhibited SARS-CoV-2 infection of human airway epithelial cells (hAEc). Oguntuyo et al. [90] reported that sera from SARS-CoV-2 naïve individuals inhibited cellular entry of SARS-CoV-2 and identified AAT as the molecule responsible. AAT strongly inhibits elastase, which also cleaves the spike protein at the S1/S2 site of a SARS-CoV-2 variant [3]. Singh and co-workers [91] generated variants of SerpinB3 — through mutagenesis of its reactive center loop — that were more potent in inhibiting TMPRSS2 than AAT. Rosendal *et al.* [92] used a three-dimensional lung cell model using primary cells from multiple donors and found that more resistant donors to *in vitro* SARS-CoV-2 infection expressed higher transcript levels of *SERPINA1* (AAT gene), *SERPINE1*, and *SERPINE2* in airway cells (basal, ciliated, club, and goblet cells) [92].

**Figure 1.**
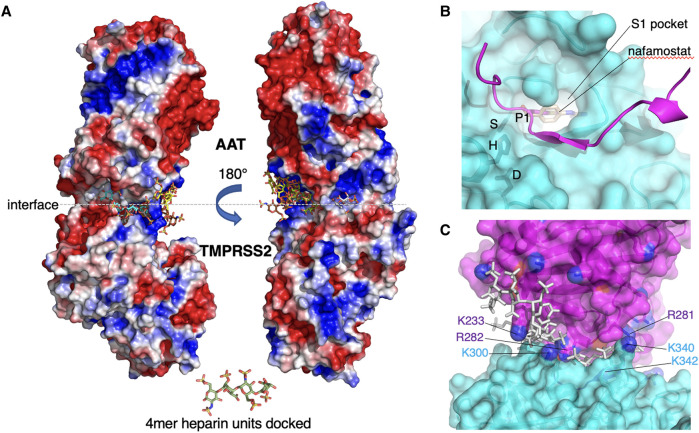
*In silico* modeling demonstrating the interaction between TMPRSS2 and AAT and how heparin stabilizes the TMPRSS2–AAT complex. **(A)** Heparin molecules (colored stick figures) stabilize TMPRSS2–AAT association by acting as electrostatic bridges. Electrostatic potential surfaces of the TMPRSS2–AAT complex (blue = positive, red = negative) showing docked heparin 4mers binding to repelling electropositive patches at the molecular interface of TMPRSS2 and AAT. **(B)** Molecular surface of TMPRSS2 (cyan) showing bound reactive center loop (RCL) of AAT (magenta cartoon). Catalytic residues are labeled. P1 sidechain of AAT occupies the S1 site of TMPRSS2, superimposed on the TMPRSS2 inhibitor nafamostat (wheat). (C) Location of electropositive Lys/Arg residues (TMPRSS2 = cyan; AAT = magenta) in the vicinity of electronegative heparin (gray sticks) binding sites at the TMPRSS2(cyan)–AAT(magenta) interface. Semi-transparent molecular surface is shown, and Lys/Arg residues that contribute to unfavorable electrostatics at the interface are shown and labeled (sidechain nitrogen atoms colored blue). AAT = alpha-1-antitrypsin; TMPRSS2 = Transmembrane Protease, Serine 2. Reproduced with permission from Springer Nature (Bai et al. [87]).

Negatively charged polysaccharides are capable of augmenting the interaction between a serpin and its cognate serine protease [87,93]. Heparin has the highest negatively charge density of any biologic molecules. We found that AAT inhibition of TMPRSS2 activity and of the intracellular burden of human coronavirus 229E (HCoV-229E) in primary hAEc were augmented significantly by enoxaparin (a low molecular mass heparin) [87]. Detailed *in silico* modeling demonstrated: (i) suboptimal electrostatic complementarity between AAT and TMPRSS2 at both the buried interface and solvent-exposed interface rim in a molecular charge model (Figure 1A); (ii) the reactive center loop of AAT (Figure 1B, magenta) adopts an inhibitory-competent conformation compared with the crystal structure of TMPRSS2 (Figure 1B, cyan) bound to an exogenous (nafamostat) or endogenous (HAI-2) inhibitors of TMPRSS2; and (iii) negatively charged heparin (stick figures in Figure 1A–C) bridges adjacent electropositive patches at the TMPRSS2–AAT interface, neutralizing otherwise repulsive forces [87].

In addition to its anticoagulant effect and its ability to augment AAT inhibition of TMPRSS2, heparin may antagonize COVID-19 by other means (Table 1). Since AAT and enoxaparin individually and synergistically antagonize several of the pathogenic mechanisms of COVID-19, we posit that they will be more effective than agents that only inhibit TMPRSS2 such as camostat [94].

**Table 1.** Biological activities of alpha-1-antitrypsin and heparin that could help ameliorate severe COVID-19

Alpha-1-antitrypsin [23,87,163]	Heparin [164-166]
Inhibits TMPRSS2	Competitively inhibits cell surface heparan sulfate, a co-receptor SARS-CoV-2
Induces autophagy, shown to be a host-protective mechanism against MERS-CoV-2	Inhibits coagulation
Inhibits inflammation by various mechanisms	Inhibits inflammation by inhibiting NFκB activation and binding to pro-inflammatory molecules (IL-8, major basic protein, and complement components)
Inhibits neutrophil elastase, which mediates acute lung injury and possibly processes the spike protein of SARS-CoV-2	Augments C1-esterase inhibitor (C1-INH) inhibition of kallikrein → decrease capillary leak
Alters morphology of neutrophil extracellular traps, which is known to induce immunothrombosis of COVID-19	Reduces the release and activity of IL-6
Inhibits thrombin	Binds to extracellular histones released from dead cells, mitigating histone-mediated endothelial and organ dysfunction
Protects endothelial cells	Interacts with endothelial cells to maintain vascular integrity

## Other activities of AAT could mitigate severity of COVID-19

**Figure 2.**
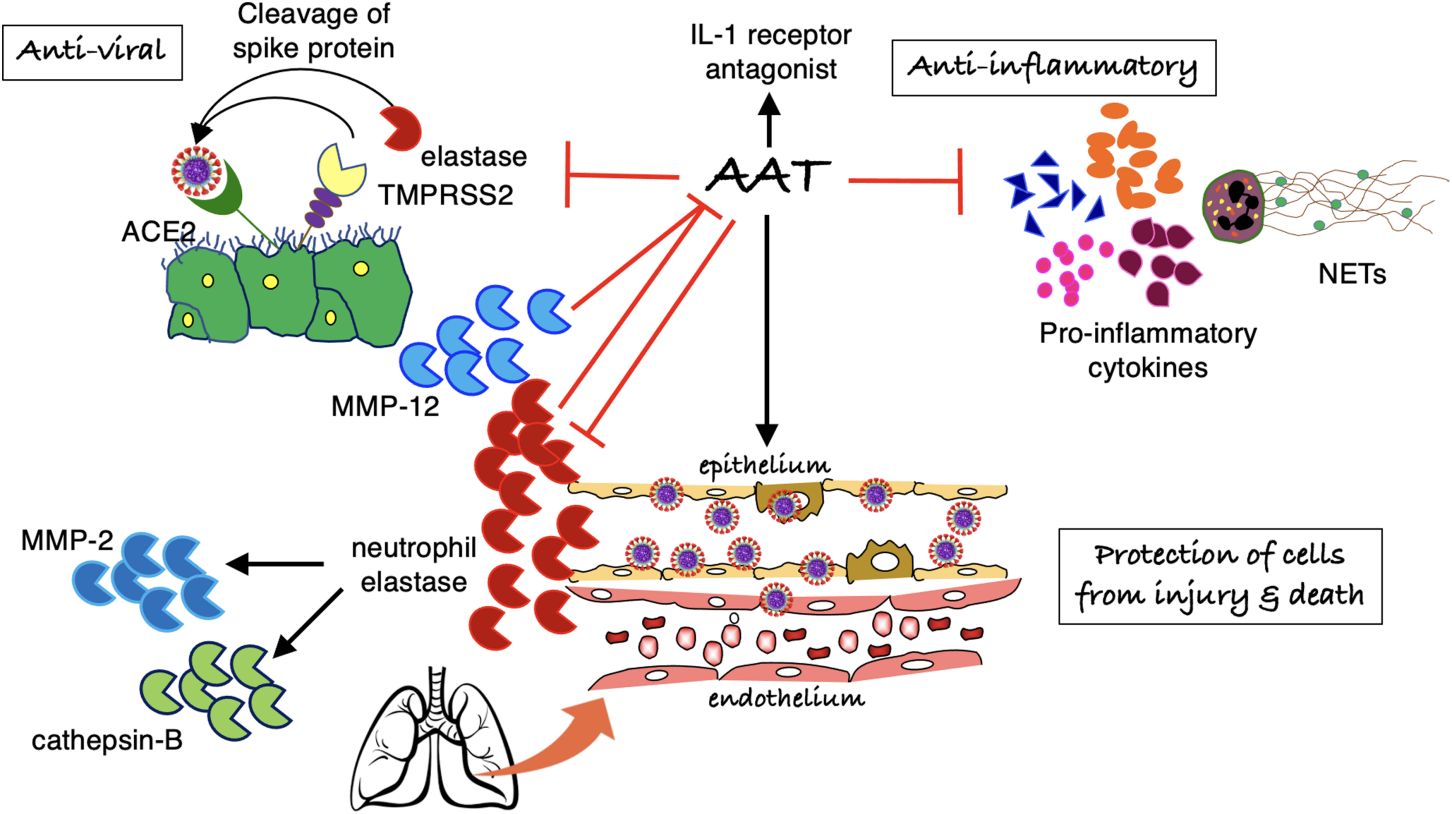
Mechanisms by which AAT may help antagonize SARS-CoV-2 and ameliorate the pathogenic mechanisms of COVID-19. AAT has: (*i*) anti-viral infection effects by inhibiting TMPRSS2 and possibly through induction of autophagy, (*ii*) anti-inflammatory effects, and (*iii*) anti-apoptotic and anti-elastase functions, protecting infected cells from injury and death. An increase in protease (neutrophil elastase, MMP-2, MMP-12) to anti-protease ratio may not only deplete AAT but elastase and MMP-12 are known to cleave AAT at a site other than the reactive center loop of AAT (denoted by inhibitory red ‘arrows’). AAT = alpha-1-antitrypsin; ACE2 = angiotensin converting enzyme 2; MMP-2 = matrix metalloproteinase-2; MMP-12 = matrix metalloproteinase-12; NETs = neutrophil extracellular traps; TMPRSS2 = Transmembrane Protease, Serine 2.

In addition to inhibiting TMPRSS2, preventing the processing of the spike protein and cellular entry of SARS-CoV-2, AAT has a panoply of other activities that could antagonize both the intracellular virus and the multiple pathogenic mechanisms associated with severe COVID-19 (Figure 2 and Table 1) [23,95]. Such host-directed activities are less likely to be affected by SARS-CoV-2 mutations.

### AAT protects against microbes including RNA viruses

AAT augments host immunity via several mechanisms against various pathogens including influenza [96], HIV [97–101], *Pseudomonas aeruginosa* [102], *Mycobacterium intracellulare* [103], and *Streptococcus pneumoniae* [104]. AAT reduced the burden of cell-associated *M. intracellulare* through induction of autophagy [103]. Since autophagy has been implicated in controlling MERS-CoV [105], AAT-induced autophagy is a plausible mechanism by which SARS-CoV-2 is killed.

### AAT has potent anti-inflammatory activities

AAT inhibits production of pro-inflammatory cytokines [63,103,106–113], induces expression of the anti-inflammatory IL-1Ra [112], and skews T cell differentiation toward the T regulatory phenotype [114]. These anti-inflammatory effects of AAT — augmented by an increase in sialylation of AAT [110] — could mitigate the injurious hyperinflammatory response typically seen seven to 10 days after the symptom onset of severe COVID-19. In contrast with the anti-inflammatory effects of glucocorticoids, AAT induces a more selective cytokine shift toward resolution; e.g. suppression of pro-inflammatory cytokines plus induction of endogenous IL-1Ra, whereas glucocorticoid causes a more global immunosuppression including inhibition of IL-1Ra expression [112]. Interestingly, when AAT and glucocorticoid are combined in macrophages, lower glucocorticoid concentrations used allowed up-regulation of IL-1Ra, whereas higher glucocorticoid levels antagonized the effects of AAT [112]. Vitamin D deficiency may also lead to decreased release of AAT from T cells, causing decreased release of the anti-inflammatory cytokine IL-10 [115,116]. Tumpara et al. [117] showed that recombinant AAT (made in Chinese hamster ovary cells) was more potent than plasma-derived AAT in inhibiting pro-inflammatory cytokine production from peripheral blood mononuclear cells stimulated with both lipopolysaccharide and spike proteins.

Recently, we reported the novel finding that AAT binds to the cytoplasmic glucocorticoid receptor (GR) in macrophages, with the AAT–GR complex having gene regulatory, anti-inflammatory, and host-defense properties [118]. Perhaps relevant to COVID-19, we found that the AAT–GR complex inhibited IL-8 production from stimulated macrophages. Since IL-8 is a potent chemokine for neutrophils and neutrophilia is a poor prognostic marker for COVID-19 [119], AAT antagonism of IL-8 production may be another mechanism against COVID-19.

### Role of proteases in acute lung injury and antagonism by AAT

AAT has been shown to protect the lungs from acute lung injury [120,121]. Since an imbalance of protease and anti-protease is implicated in the pathogenesis of COVID-19 acute lung injury, absolute or relative deficiency of functional AAT is a plausible risk factor for severe COVID-19 [122,123]. In 39 critically ill patients with COVID-19, the median AAT level was modestly increased (by 1.5-fold the upper limits of normal), but there was a disproportionately increase in blood matrix metalloproteinase-12 (MMP-12, produced mainly by macrophages) and neutrophil elastase by ≥10-fold and four- to seven-fold, respectively [124]. Furthermore, the peak MMP-12 and neutrophil elastase levels were higher in those who subsequently died. When AAT phenotype was analyzed temporally, there was an increase in sialylation of the M0 and M1 AAT glycoforms in all those who died and in 59% of those who survived, suggesting that a shift in sialylation was a counterregulatory response to increase anti-elastase activity in these critically ill COVID-19 patients [124]. Interestingly, the increased protease:anti-protease ratio seen with COVID-19 may be due to cleavage of AAT by proteases, as evinced by increased AAT fragments C-36 (cleavage product of neutrophil elastase) and C-42 (cleavage product of MMP-12) (Figure 2) [125]. Additionally, cathepsin B may play a role in the hyperinflammatory response of COVID-19 [126] and AAT inhibits neutrophil elastase induction of cathepsin B *in vivo* and *in vitro* (Figure 2) [127]. Elastase also induces MMP-2, the plasma levels of which correlated with in-hospital death in patients with severe COVID-19 [128]. Through AAT inhibition of neutrophil elastase, subsequent inhibition of MMP-2 production is another mechanism by which AAT antagonizes injury to the lung extracellular matrix (Figure 2) [127,129]. AAT also has been shown to have anti-apoptotic function in epithelial and endothelial cells [130,131] (Figure 2), which may not be necessarily dependent on its anti-elastase activity as has been shown for protection against hepatocellular death [132]. In assessing lung function 12 months after recovery from COVID-19, those with PiMS and PiMZ genotypes had lower AAT levels (compared with PiMM), especially in those with persistently impaired lung function [133].

In 31 patients with SARS-CoV-2 infection, plasma levels of exosome-associated neutrophil elastase were elevated compared with control subjects and positively correlated with signs of endothelial damage and circulating AAT levels [134]. Whereas AAT was able to inhibit purified neutrophil elastase in macrovascular endothelial cells, exosome-associated neutrophil elastase was resistant to AAT inhibition.

### AAT inhibition of ADAM17 antagonizes several pathogenic mechanisms of COVID-19

**Figure 3.**
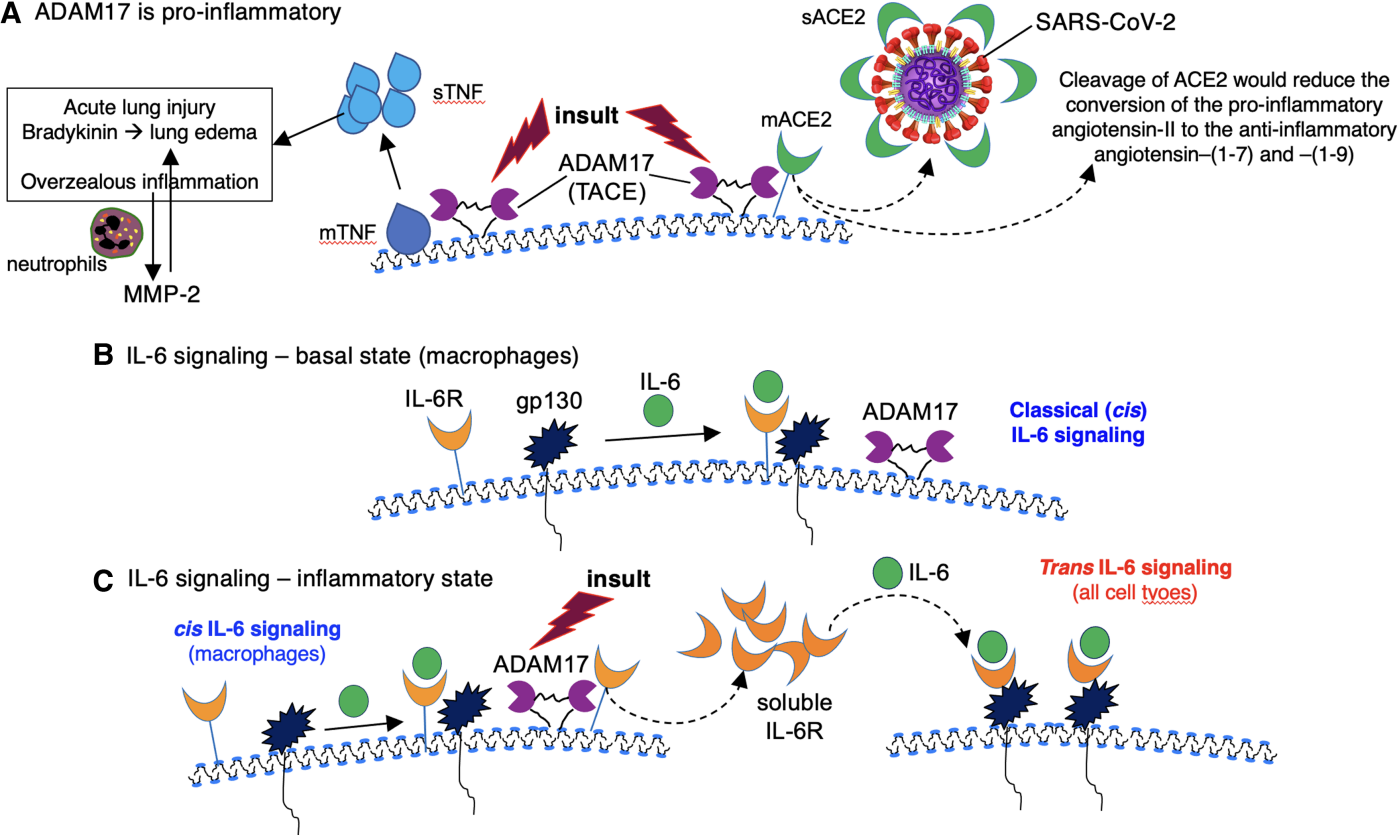
Mechanisms by which ADAM17 may contribute to the hyperinflammatory pathogenesis of COVID-19. (**A**) ADAM17, also known as TNF converting enzyme, is able to cleave membrane-bound TNF (mTNF) to soluble TNF (sTNF), enhancing the inflammatory and injurious response such as acute lung injury and edema. In addition, the inflammatory response induces MMP-2 in neutrophils, causing further injury to the lung extracellular matrix. Activated ADAM17 is also cleaves mACE2 to soluble ACE2 (sACE2). This process may enhance inflammation by preventing mACE2 from catalyzing pro-inflammatory angiotensin to the less inflammatory metabolites (angiotensin–(1–7) and angiotensin–(1–9)). While soluble ACE2 is able to bind free virus, reduction in mACE2 increases both inflammation and egress of newly synthesized viral particles to infect other cells. (**B**) During the basal state, IL-6 signals by first binding to membrane-bound IL-6R (on macrophages), followed by engagement of the IL-6-IL-6R complex with gp130, which contains an intracellular signaling moiety that IL-6R lacks. The classical type of IL-6 signaling is known as *cis*-IL-6 signaling. (**C**) During an inflammatory state (insult), the cell surface protein ADAM17 is activated and cleaves membrane-bound IL-6R (mIL-6R) from the cell surface. The soluble IL-6R (sIL-6R) binds to IL-6 and the complex can directly bind to gp130 on all cell types to provide another mode of signaling that is more widespread and known as *trans*-IL-6 signaling. ACE2 = angiotensin converting enzyme 2; ADAM17 = a disintegrin and metalloprotease 17; IL-6 = interleukin-6; IL-6R = IL-6 receptor; MMP2 = matrix metalloproteinase-2; TNF = tumor necrosis factor.

ADAM17 is a cell surface protease that causes shedding of ACE2, the cell surface receptor for SARS-CoV-2 [135] (Figure 3A). While this cleavage would theoretically reduce initial binding SARS-CoV-2 to the cell membrane, reduction of membrane-bound ACE2 (mACE2) may increase lung injury and edema because ACE2 has been shown to protect the lungs from injury — by reducing both bradykinin production and neutrophil infiltration as well as catalyzing angiotensin II to the anti-inflammatory angiotensin-(1–7) and angiotensin-(1–9); both metabolic products of angiotensin II also protect lung epithelial cells from death [136–138]. ADAM17, aka TNF converting enzyme, also cleaves membrane-bound TNF to its soluble form, perpetuating the inflammatory response including the induction of MMP-2 (Figure 3A).

Both membrane-bound and soluble IL-6R have pro-inflammatory function but the former occurs mostly in macrophages, whereas the latter occurs in all cell types (Figure 3B,C). Upon binding of IL-6 to membrane-bound IL-6R, the IL-6–IL-6R complex engages gp130, a cell surface molecule that transmits downstream intracellular signaling, a process known as *cis*-IL-6 signaling (Figure 3B,C, left-hand side). Soluble IL-6R binding to IL-6 can also bind to gp130, a process known as *trans*-IL-6 signaling (Figure 3C, right-hand side). During inflammatory states, ADAM17 is activated and cleaves membrane forms of IL-6 and IL-6R to their soluble forms [139–141], resulting in more widespread IL-6-mediated inflammation (Figure 3C). ADAM17 also induces shedding of fractalkine, a membrane-bound chemokine that facilitates intercellular adhesion and mainly found in neurons and endothelial cells [142]; thus, ADAM17 may reduce intercellular adhesion between endothelial cells.

AAT inhibits activity of ADAM17 [106,132,143]. Given that ADAM17 may augment inflammation and other mechanisms that promote lung injury, AAT inhibition of ADAM17 is likely to be anti-inflammatory and to protect the lungs from injury. While AAT inhibition of ADAM17 could also preserve ACE2 on the cell surface, such preservation of the receptor for SARS-CoV-2 may paradoxically decrease viral infectivity to other cells by decreasing the egress of newly synthesized viral particles [138,144].

### The role of neutrophil extracellular traps in the immunothrombosis of severe COVID-19 and possible antagonism by AAT

Elevated absolute neutrophil number, increased neutrophil percentage, and high neutrophil:lymphocyte ratio in the blood predict progression to severe COVID-19 [119]. One study showed that neutrophil:lymphocyte ratio ≥4.5 and ≥6.5 were associated with significantly increased severity and mortality, respectively [145]. There is also increasing evidence that aberrant formation of neutrophil extracellular traps (NETs) — comprised of neutrophil-derived decondensed chromatin decorated with elastase, cathepsin G, and histones — play a pathogenic role in the immunothrombosis, mucous secretions, and cytokine production seen with severe COVID-19 [146-152]. In hospitalized patients with COVID-19, increased NETs formation was found in the plasma, tracheal secretions, and lung tissues [152]. In post-mortem lung tissues of individuals who died from COVID-19, NETs were found in the airways, interstitium, and arterioles [151]. Since elastase plays a key role in NETs formation by degrading specific histones and promoting chromatin decondensation [153], AAT has the potential to inhibit pathogenic NETs formation [154]. This notion is supported by an *ex vivo* pre-COVID-19 study showing that AAT is able to change the shape and adherence of NETs [155]. However, in the bronchoalveolar lavage fluid of COVID-19 patients, it was found that AAT–elastase complex was not found (due to elastase binding to acute phase proteins, histones, and C3) and that exogenous AAT did bind to NETs but did not qualitatively inhibit NETs formation [156].

## Current evidence for AAT treatment of COVID-19

AAT is presently administered to patients with confirmed AAT deficiency and airflow limitation [82]. A patient survey of AAT-deficient patients showed that AAT replacement reduced the number and severity of lung infections [157]. In 19 AAT-deficient emphysema patients in whom AAT was abruptly discontinued due to insurance reimbursement issues, the number of pulmonary exacerbations — historically often due to viral or bacterial infections — dramatically rose [158].

A 43-year-old woman with cystic fibrosis, who developed severe respiratory deterioration with marked increase in pro-inflammatory cytokines in the airways and the blood following acquisition of COVID-19, was administered intravenous AAT at 120 mg/kg/week for four consecutive weeks [159]. Following each AAT dose, both plasma and airway IL-1β, IL-6, and IL-8 levels decreased and airway neutrophil elastase level decreased, accompanied by clinical and radiographic improvements [159].

An open-label study administered AAT to nine patients requiring hospitalization for mild or moderate COVID-19 — four received inhaled AAT at 100 mg/day for 7 days while five received inhaled AAT at the same dose plus intravenous AAT at 60 mg/kg on Days 1, 3, and 5 [88]. Two of the patients also received remdesivir and two received dexamethasone. In all nine patients, the oxygenation improved, serum C-reactive protein decreased, and all were discharged in good functional status. While there was no control group in this study, an analysis of a contemporaneous observational cohort (CORSAAR) of 23 COVID-19 patients matched for age and disease severity revealed three COVID-19 deaths and a delayed decrease in C-reactive protein in the historical ‘control’ group that did not receive exogenous AAT [88]. Three Israeli patients who were critically ill with COVID-19 (all mechanically ventilated) were administered three doses of intravenous AAT (1–2 days apart) early in the hospitalization (initiated Day 2–7 of hospitalization) and all improved objectively (as measured by the SOFA score and PaO_2_/FiO_2_ ratio) within a day of administering the AAT; a fourth patient who received only one dose of AAT at ∼Day 21 of hospitalization succumbed [160]. In a phase 2, multi-center, randomized, double-blind, placebo-controlled trial of 36 patients with moderate to severe COVID-19-associated acute respiratory distress syndrome who received either weekly placebo (*n* = 11), weekly intravenous AAT at 120 mg per kg (*n* = 13), or AAT once followed by weekly placebo (*n* = 12), AAT treatment (in the two latter groups) resulted in significant decrease in IL-6 compared with the placebo group at 1 week [161]. However, AAT administration (*n* = 25) also reduced soluble receptor for TNF [161], which may increase free TNF. While there was no difference in mortality or ventilator-free days, the AAT-treated patients had a trend toward decreased time on the ventilator *P* = 0.44 [161]. Strassmair and Stangl [162] have proposed the idea that delivery of mesenchymal stem cells that express AAT, with their ability to ‘home-in’ to the lungs, might be an effective mechanism to deliver AAT.

## Conclusion

Several large studies demonstrate robust epidemiologic association between AAT deficiency and increased COVID-19 infection, hospitalization, severe disease, and mortality. Enoxaparin augments AAT inhibition of TMPRSS2 activity and thus may synergize with AAT in inhibiting SARS-CoV-2 entry of cells. Because both AAT and enoxaparin have biological properties that antagonize several pathogenic mechanisms of COVID-19, this pharmaceutical combination is promising for those critically ill from recalcitrant COVID-19. Thus, future translational investigations and clinical trials should be undertaken with combined AAT and enoxaparin in patients who are severely ill with COVID-19.

## Perspectives

- While there are effective vaccines against the prevention of severe COVID-19, treatment for patients who are critically ill with COVID-19 remains suboptimal.
- Based on the emerging evidence that AAT inhibits SARS-CoV-2 infection and many of the pathogenic mechanisms of COVID-19, a review of this topic is timely.
- Several large studies demonstrate robust epidemiologic association between AAT deficiency and increased COVID-19 infection, hospitalization, severe disease, and mortality. Enoxaparin, a low molecular mass heparin, augments AAT inhibition of TMPRSS2 activity and thus may synergize with AAT in inhibiting SARS-CoV-2 entry of cells. Because both AAT and enoxaparin have biological properties that antagonize several pathogenic mechanisms of COVID-19, this pharmaceutical combination is promising for those critically ill from recalcitrant COVID-19. Thus, future translational investigations and clinical trials should be undertaken with combined AAT and enoxaparin in patients who are severely ill with COVID-19.

## Abbreviations

AAT: alpha-1-antitrypsin
ACE2: angiotensin converting enzyme 2
COPD: chronic obstructive pulmonary disease
GR: glucocorticoid receptor
GWAS: genome-wide association study
hAEc: human airway epithelial cells
IL-1: interleukin-1
IL-6R: IL-6 receptor
MAF: minor allele frequencies
MMP-12: metalloproteinase-12
PiMM: protease inhibitor MM
TMPRSS2: transmembrane protease 2
TNF: tumor necrosis factor
